# RSK-3 promotes cartilage regeneration via interacting with rpS6 in cartilage stem/progenitor cells

**DOI:** 10.7150/thno.44875

**Published:** 2020-05-25

**Authors:** Shuai Zhang, Md Rana Hamid, Ting Wang, Jinqi Liao, Liru Wen, Yan Zhou, Pengfei Wei, Xuenong Zou, Gang Chen, Junhui Chen, Guangqian Zhou

**Affiliations:** 1 Department of Medical Cell Biology and Genetics, Guangdong Key Laboratory of Genomic Stability and Disease Prevention, Shenzhen Key Laboratory of Anti-aging and Regenerative Medicine, and Shenzhen Engineering Laboratory of Regenerative Technologies for Orthopaedic Diseases, Health Sciences Center, Shenzhen University, Shenzhen 518060, China.; 2 Lungene Technologies, B606, Yinxing Scientific Building, Lonhua District, Shenzhen, 510086, China.; 3 Department of Internal Medicine, General Hospital of Shenzhen University, Shenzhen University, Shenzhen 518060, China.; 4 Department of Spine Surgery, Orthopedic Research Institute, Guangdong Provincial Key Laboratory of Orthopedics and Traumatology, The First Affiliated Hospital of Sun Yat-sen University, Guangzhou 510080, China.; 5 Jiangxi Provincial People's Hosptial Affiliated to Nanchang University, Nanchang, 330006, Jiangxi, China.; 6 Intervention and Cell Therapy Center, Peking University Shenzhen Hospital, Shenzhen Peking University-The Hong Kong University of Science and Technology Medical Center, Shenzhen 518035, Guangdong, China.

**Keywords:** Cartilage stem/progenitor cell, MRL/MpJ, osteoarthritis, RSK-3, STR/Ort

## Abstract

**Rationale:** Cartilage stem/progenitor cells (CSPC) are a promising cellular source to promote endogenous cartilage regeneration in osteoarthritis (OA). Our previous work indicates that ribosomal s6 kinase 3 (RSK-3) is a target of 4-aminobiphenyl, a chemical enhancing CSPC-mediated cartilage repair in OA. However, the primary function and mechanism of RSK-3 in CSPC-mediated cartilage pathobiology remain undefined.

**Methods:** We systematically assessed the association of RSK-3 with OA in three mouse strains with varying susceptibility to OA (MRL/MpJ>CBA>STR/Ort), and also RSK-3^-/-^ mice. Bioinformatic analysis was used to identify the possible mechanism of RSK-3 affecting CSPC, which was further verified in OA mice and CSPC with varying RSK-3 expression induced by chemicals or gene modification.

**Results:** We demonstrated that the level of RSK-3 in cartilage was positively correlated with cartilage repair capacities in three mouse strains (MRL/MpJ>CBA>STR/Ort). Enhanced RSK-3 expression by 4-aminobiphenyl markedly attenuated cartilage injury in OA mice and inhibition or deficiency of RSK-3 expression, on the other hand, significantly aggravated cartilage damage. Transcriptional profiling of CSPC from mice suggested the potential role of RSK-3 in modulating cell proliferation. It was further shown that the in vivo and in vitro manipulation of the RSK-3 expression indeed affected the CSPC proliferation. Mechanistically, ribosomal protein S6 (rpS6) was activated by RSK-3 to accelerate CSPC growth.

**Conclusion:** RSK-3 is identified as a key regulator to enhance cartilage repair, at least partly by regulating the functionality of the cartilage-resident stem/progenitor cells.

## Introduction

Articular cartilage is an avascular tissue with chondrocyte as the singular cell type 1. Traumatic or progressive degeneration of articular cartilage disrupts its integrity and leads to joint diseases such as osteoarthritis (OA), which often progresses with aging and has become a leading cause of disability in elder populations 2. The declined cellularity is commonly observed throughout the whole OA progression, including improper proliferation, differentiation and dedifferentiation, senescence, autophagy, and apoptosis 3. On the other hand, although considered as low- or non-self-renewing and lacking reparative ability, multiple studies have shown that articular cartilage contains stem/progenitor cell populations with typical surface markers of mesenchymal stem cells found in many other adult tissues, which may be the core cell subpopulations contributing to the maintenance of tissue homeostasis and in the meanwhile the target of OA pathological factors 1. Nowadays, although exogenous stem cells are widely studied for cartilage repair 4-7, these endogenous cartilage-resident stem/progenitor cells (CSPC) are also a promising cell source for regenerative medicine of cartilage, and novel factors and compounds activating these cells are emerging 8-10. CSPC are usually identified by their expression of CD44, CD29, CD90 and CD105 markers 11-13. In simplicity, CD44^+^ CD90^+^ CSPC displays a number of characteristics of stem cells and can be referred to as cartilage stem cells 14,15. However, the origin and functional properties of CSPC and their molecular regulation are still poorly understood.

STR/Ort mice spontaneously develop OA early in life, with cartilage degeneration of the joint similar to that present in human OA 16,17. The degenerative changes of knee joints in STR/Ort mice progress from the initial lesion to eburnation over a period of 4-8 months 18. Mild lesions are often observed in one or other knee joint at 10 weeks of age 19. The steadily increasing OA incidence and severity are from 18 weeks of age 16, 20. By 20 weeks most STR/Ort mice have cartilage injury of the knees 19. Our previous study indicates that STR/Ort mice at 4-month age exhibit cartilage degeneration and lesion in the knee joints, which are aggravated in 8-month-age mice 12. Although the age of onset and the severity of cartilage lesions are variable in different studies, most of the studies suggest the steadily increasing lesion of knee cartilage occurs in 5-month-old STR/Ort mice.

By contrast, MRL/MpJ mice exhibit an exceptional ability for wound repair and tissue regeneration, including that of the articular cartilage and skin 21. Following surgery-induced destabilization of the medial meniscus (DMM), MRL/MpJ mice show remarkable cartilage healing ability, suggestive of an intrinsic regenerative capacity of the articular cartilage 22-24. The difference in repair between the two mouse models provides an opportunity to examine the molecular mechanisms underlying cartilage repair. We recently report that CSPC from STR/Ort mice exhibit lower proliferative and differential capacity than that from the control CBA mice, highlighting that multiple signal pathways are involved in CSPC regulation 12. Preliminary study also suggests that the MAPK signaling may be involved in the CSPC function.

The ribosomal s6 kinase (RSK) family is highly conserved Ser/Thr kinase that regulates diverse cellular processes, such as cell growth, motility, survival and proliferation, and is the downstream effector the ERK1/2 and other MAPK pathways 25, 26. In turn, RSK-3 has been shown to regulate various molecules such as CREB, SRF, FOS, rpS6, ER81, ERα, NF-κB, NFATc4, NFAT3, the transcription initiation factor TIF1A, and eIF4B 25,27. Our previous work indicates that 4-aminobiphenyl (4-ABP) promotes CSPC-mediated cartilage regeneration in OA mice via modulating the RSK-3 function 28, suggesting that RSK-3 may be a key regulator of CSPC in OA progress, particularly via its function in CPSC.

Herein, we further investigate the role of RSK-3 in CSPC and their effects on cartilage degeneration and regeneration by using RSK3-deficient mice and mouse models with varying capability of cartilage repairing.

## Methods

### Animals

Male MRL/MpJ mice, CBA mice (Model Animal Research Center of Nanjing University, Nanjing, China), STR/Ort mice (Shanghai Research Center for Model Organisms, Shanghai, China), C57BL/6J mice (Guangdong Medical Laboratory Animal Center, Guangzhou, China) and global RSK-3 knockout (RSK3^-/-^) mice congenic on a C57BL/6J background (Cyagen Biosciences, Guangzhou, China) were maintained in polypropylene cages and housed under standard conditions.

### Surgery-induced osteoarthritis and chemical treatment

An OA model was created in mice by surgical DMM, according to a previously reported protocol 29. Briefly, male mice were anesthetized with 2% isoflurane in air using an anesthesia machine (RWD Life Science Co., Ltd., Shenzhen, China). After opening the right knee joint capsule, the medial meniscotibial ligament, which anchors the medial meniscus to the tibial plateau, was cut to destabilize the joint. Cartilage injury beneath the medial meniscus was avoided. The knee joint capsule was then closed with a suture. In the sham group, the knee joint capsule was opened without further intervention. The surgery was performed by the same operator who was skilled at DMM operation and blinded to this study. After the surgery, the mice were randomized to allocate to different experimental groups. Following surgery (1 or 2 months), the knee joints were collected for toluidine blue staining and Osteoarthritis Research Society International (OARSI) scoring as previously described 30. All data analysis was performed in a double-blind manner by two investigators who were given no information about the experimental groups.

In our previous work, 4-ABP was found to promote RSK-3 expression 28. To assess the role of regulating RSK-3 by the chemicals in the OA model, DMM was created in male C57BL/6J mice and then 10 μM 4-ABP (MedChemExpress, USA) or RSK-3 inhibitor (BI-D1870, MedChemExpress, USA) formulated in saline was immediately administered by intra-articular injection (4 μL) once a week for 4 weeks, and the knee joints were collected for safranin O-fast green staining. OARSI scores were used for lesion assessment.

### CSPC isolation and RNA-sequencing

Knee joints from the normal STR/Ort, CBA and MRL/MpJ mice (5 months old; n = 3 per group) were isolated, and the femoral and tibial cartilage was dissected under a stereoscopic microscope (Ruihoge, China). The collected cartilage was washed with Versene solution (Invitrogen, USA) and then digested in DMEM/F-12 medium containing 0.2% collagenase NB4 (SERVA, Germany), 0.2% collagenase II, 0.2% collagenase I (Life Technologies, USA), 5% FBS (SeraBest, PAN-Systech GmbH, Germany), 50 U/ml penicillin and 50 μg/ml streptomycin (Invitrogen, USA) at 37°C for 6 h with shaking. The digested material was washed twice with DMEM/F12 medium (Invitrogen, USA) and filtered through a 40-μm nylon mesh (BD Falcon, Germany). The cells were incubated with FITC-conjugated CD44 and PE-conjugated CD90 antibodies (BD Biosciences, USA) at 4°C for 30 min, and the single cell was sorted into a 96-well plate using a FACS Arila II flow cytometer (BD Biosciences, USA). Subsequently, seven CD44^+^ CD90^+^ CSPCs in each group were stored at -80°C until RNA extraction. The remaining CD44^+^ CD90^+^ CSPCs were cultured in DMEM/F12 medium with 15% FBS, 100 U/ml penicillin and 100 μg/ml streptomycin for further experiments.

Human CD44^+^ CD90^+^ CSPC were sorted from previously prepared chondrocytes isolated from a patient with OA and a patient without OA 12. The use of human specimens was approved by the regional committee for medical research ethics and the Human Ethics Committee of Shenzhen BaoAn People Hospital, and written consent was obtained from each subject.

RNA extraction, sequencing and bioinformatic analyses were performed by BGI (Shenzhen, China) according to standard procedures and as previously described 12,31. In this project, 21 samples were sequenced on Illumina HiSeq Platform with about 7 Gb data production per sample. RNA integrity was assessed using the RNA Nano 6000 Assay Kit of the Bioanalyzer 2100 system (Agilent Technologies, CA, USA). RNA quality control, read mapping and bioinformatic analysis were performed by standard bioinformatic methods 12,31. The levels of expression for each gene were normalized to fragments per kilobase of exon model per million mapped reads (FPKM). Data available at https://www.ncbi.nlm.nih.gov/sra/PRJNA598202, accession number PRJNA598202.

### Cell proliferation

Cell viability was assayed using a Cell Counting Kit-8 (CCK-8) kit (MedChemExpress, USA). Briefly, 3,000 cells were seeded in 96-well plates. Following culture for 0, 1, 2 and 3 days, CCK-8 solution was added to each well and incubated at 37°C for 1 h. The optical density at 450 nm was determined using a microplate reader (Multiskan GO, Thermo Scientific, Germany).

### Real-time PCR

Total RNA was extracted from cells using RNAiso Plus reagents (TAKARA, China), according to the manufacturer's instruction. Total RNA was transcribed into cDNA using a PrimeScript RT Master Mix kit (TransGen Biotech, China). The real-time quantitative PCR reaction was performed using a TransStart Tip green qPCR SuperMix kit (TransGen Biotech, China) in an ABI 7500 Real Time PCR system (Applied Biosystems, USA). Primer sequences were detailed in Table s1 and s2. The β-actin gene was amplified separately as an internal control to normalize for specific gene expression in the samples. The fold change was calculated using the 2^-∆∆Ct^ method.

### Short hairpin (sh) RNA-mediated gene knockdown

Mouse *Rps6ka2* or *rpS6* gene shRNA sequences were designed according to the cDNA sequence using online software (http://bioinfo.clontech.com/rnaidesigner/frontpage.jsp). Following synthesis and annealing, four double-stranded oligonucleotides (dsOligo) were cloned into the pDC316-gfp-U6 plasmid (Miaoling Bioscience & Technology, Wuhan, China), and the sequences were confirmed by PCR and DNA sequencing. Real-time PCR and western blotting were used to screen the most effective pDC316-gfp-*Rps6ka2*-shRNA or pDC316-gfp-*rpS6*-shRNA plasmid in HEK293 cells (ATCC, USA), and the most effective plasmid was packaged into a recombinant adenovirus AD-*Rps6ka2*-shRNA or AD-*rpS6*-shRNA with adenovirus packing materials in HEK293 cells. The adenovirus titer was determined using a hole-by-dilution titer assay. The silencing effect of AD-*Rps6ka2*-shRNA in CSPCs of MRL/MpJ mice or AD-*rpS6*-shRNA in CSPCs of CBA mice was validated by western blotting.

### Lentiviral RSK-3 overexpression in CSPC

The sequence of cDNA for mouse *Rps6ka2* was cloned into the Ubi-MCS-SV40-EGFP-IRES-puromycin vector, which were constructed by GeneChem Co., Ltd. (Shanghai, China). The Ubi-MCS-SV40-EGFP-IRES-puromycin lentiviral vectors were used as controls. Lentiviral particles were generated by transfecting the expression vector Ubi-MCS-SV40-EGFP-IRES-puromycin and ViraPower Packaging Mix into 293T cells according to the Invitrogen ViraPower Lentiviral Expression Systems protocol. CD44^+^ CD90^+^ CSPC was obtained from STR/Ort mice by flow cytometry sorting. The CSPC (1×10^6^ cells, passage 3) were infected with control or RSK-3-overexpressing lentiviruses (Shanghai GeneChem) for 24 h at 37°C in the presence of 4 mg/ml polybrene. Stably transfected clones for RSK-3 were validated by observing GFP expression and western blotting analysis.

### Immunochemistry and immunofluorescence

The knee joints were fixed in 4% paraformaldehyde for 24 h, and decalcified in 10% EDTA (pH 7.4) for 14 days before being embedded in paraffin. The paraffin-embedded tissue was cut into 5-μm-thick sections. The articular sections were treated with pepsin (0.25 mg/ml, Sigma) for antigen retrieval. Endogenous peroxidase activity was blocked with 3% hydrogen peroxide (for immunohistochemistry). The cells were fixed with 4% paraformaldehyde. The cells and sections were then blocked with 5% bovine serum albumin (BSA, Sigma, USA) and incubated overnight with rabbit-anti Collagen X (Abcam, 1:200, ab58632), rabbit-anti MMP-13 (Abcam, 1:200, ab39012), rabbit-anti RSK-3 (Proteintech, 1:200, 14446-1-AP), rabbit-anti p-RSK-3 (RD systems, 1:200, AF893), rabbit-anti CD44 (Abcam, 1:200, ab157107), mouse-anti CD90 (Abcam, 1:200, ab225), or rabbit-anti Ki67 (Abcam, 1:200, ab15580) diluted in 3% BSA. Finally, the cells and sections were incubated with Alexa Flour 488/546-conjugated donkey anti-rabbit/mouse secondary antibody (Invitrogen, USA) for immunofluorescence or HRP goat anti-rabbit/mouse secondary antibodies (KPL, USA) for immunochemistry. Diaminobenzidine tetrahydrochloride (ZSGB-Bio, China) was used for immunochemical staining. Images were captured under a microscope (Olympus BX51, Japan) and quantitative analysis was conducted in a blinded manner using Image-Pro Plus 6.0 software.

### Western blotting

Total protein was obtained by lysing cells in RIPA buffer containing protease and phosphatase inhibitors. Following mixing with loading buffer and boiling, the prepared samples were separated on 10% or 8% SDS-PAGE gels and transferred onto PVDF membranes (Millipore, CA, USA). After blocking with 3% BSA, the membranes were incubated overnight at 4°C with the following primary antibodies: rabbit-anti RSK-3 (Proteintech, 1:500, 14446-1-AP), rabbit-anti p-RSK-3 (RD system, 1:500, AF893), rabbit-anti mTOR (Proteintech, 1:500, 20657-1-AP), rabbit-anti p-mTOR (Abcam, 1:500, ab109268), rabbit-anti rpS6 (Abcam, 1:500, ab40820), rabbit-anti p-rpS6 (Abcam, 1:500, ab12864), rabbit-anti p-AKT (Cell Signaling Technology, 1:500, 9271), rabbit-anti AKT (Cell Signaling Technology, 1:500, 9272), or mouse-anti β-actin (Abcam, 1:2000, ab8226). The membranes were then washed in TBS-Tween 20 and incubated with horseradish peroxidase-coupled anti-rabbit or anti-mouse antibodies (KPL, MD, USA), and were detected by chemical luminescence and visualized on a luminescent image analyzer (ImageQuant LAS4000mini, Sweden). Densitometric analyses were performed using Gel-Pro Analyzer 4.0 software (Media Cybernetics, Rockville, USA).

### Statistical analyses

All data were expressed as the mean ± SD. Differences between groups were assessed by independent-samples t-test or by one-way analysis of variance (ANOVA) followed by Tukey's Multiple Comparison test. All analyses were performed in SPSS 22.0 software and *p* < 0.05 was considered statistically significant.

## Results

### Different mouse strains exhibit varying capacities for cartilage repair

**Figure 1:**
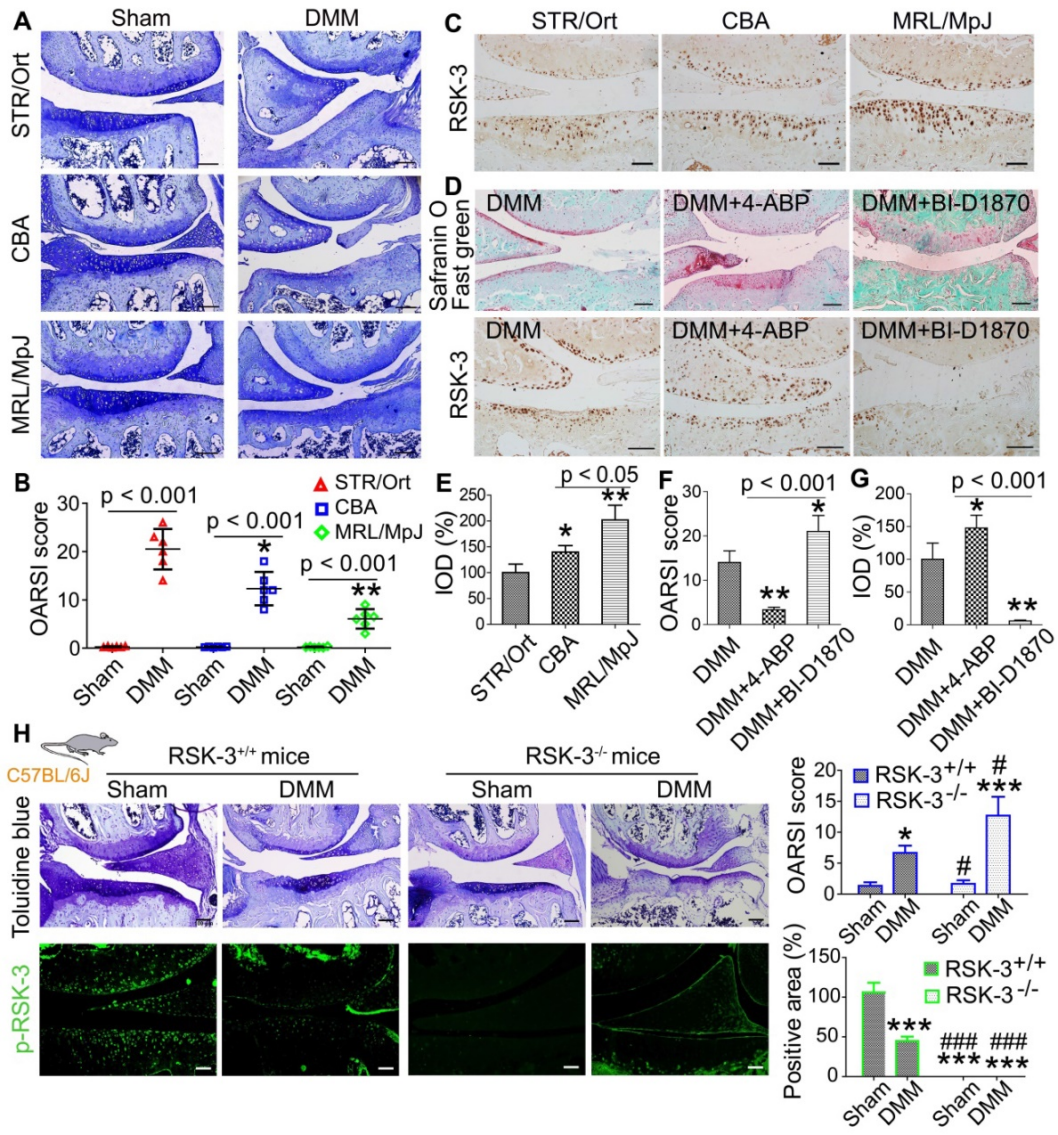
** RSK-3 promote cartilage repair.** (**A**) Representative images of toluidine blue staining in STR/Ort, CBA and MRL/MpJ articular cartilage subjected to destabilization of medial meniscus (DMM)-induced osteoarthritis injury or sham procedure. (**B**) OARSI scores for osteoarthritis cartilage histopathological assessment, *p < 0.05, **p < 0.01 versus STR/Ort (n = 6 mice per group). (**C**) Expression levels of RSK-3 in the articular cartilage of normal 5-month age STR/Ort, CBA, MRL/MpJ mice. (**D**) Safranin-O/fast green and RSK-3 staining in knee joints of C57/BL6J mice subjected to DMM injury and 4-aminobiphenyl (4-ABP) or BI-D1870 for 4 weeks. *p < 0.05, **p < 0.01 versus DMM (n = 6 per group). (**E-G**) Statistical analysis of the data shown in (C,D). *p < 0.05, **p < 0.01 versus STR/Ort (n = 6 per group). (H) Toluidine blue staining and immunolabeling of p-RSK-3 in articular cartilage from RSK-3^+/+^ and RSK-3^-/-^ mice subjected to DMM injury or sham procedure. *p < 0.05, ***p < 0.001 versus sham (RSK-3^+/+^), ^#^*p* < 0.05, ^###^*p* < 0.001 versus DMM (RSK-3^+/+^) (n = 6 per group). Scale bars = 100 µm.

A previous study showed that the relatively non-invasive procedure of monitoring ear-hole closure broadly predicted tissue regenerative capacity in mammals 32. Therefore, we first conducted a 2-mm ear punch assay to indirectly assess whether STR/Ort, CBA and MRL/MpJ mice may have different capacities for tissue repair and wound healing (Figure s1A). After three months, we found that the ear holes in MRL/MpJ mice were almost fully closed, whereas the ear holes in CBA and STR/Ort mice had not fully healed by this time point (Figure s1B,C). We inferred that these results may indicate differences in cartilage repair ability. Our previous report indicates that STR/Ort mice at 8 months of age have markedly degenerative lesion of knee cartilage compared with age-matched CBA mice 12. The current results also confirmed the spontaneous cartilage degradation of 8-month-old STR/Ort mice and the intact cartilage was shown in CBA and MRL/MpJ mice without obvious cartilage degradation (Figure s2). Under DMM-induced OA, cartilage in MRL/MpJ mice exhibited a milder OA severity compared with STR/Ort and CBA mice, as assessed by OARSI score (Figure 1A,B). These data support the different repair capacities of these three murine species.

### RSK-3 promotes cartilage repair in OA mice

To characterize the levels of RSK-3 in these three murine strains, RSK-3 expression was determined in the joint cartilage of 5-month-old mice and the expression of RSK-3 was highest in the cartilage from MRL/MpJ mice (Figure 1C,E), which suggested the positive association of RSK-3 level and cartilage repair. Further evidences showed that enhancing RSK-3 expression by 4-ABP markedly attenuated OA injury in C57BL/6J mice (Figure 1D,F,G). Inhibition or deficiency of RSK-3 expression significantly aggravated cartilage damage in DMM induced OA changers in C57BL/6J mice (Figure 1D,F-H). These data support that RSK-3 is a key molecule enhancing cartilage repair.

### RSK-3 stimulates the CSPC proliferation

**Figure 2:**
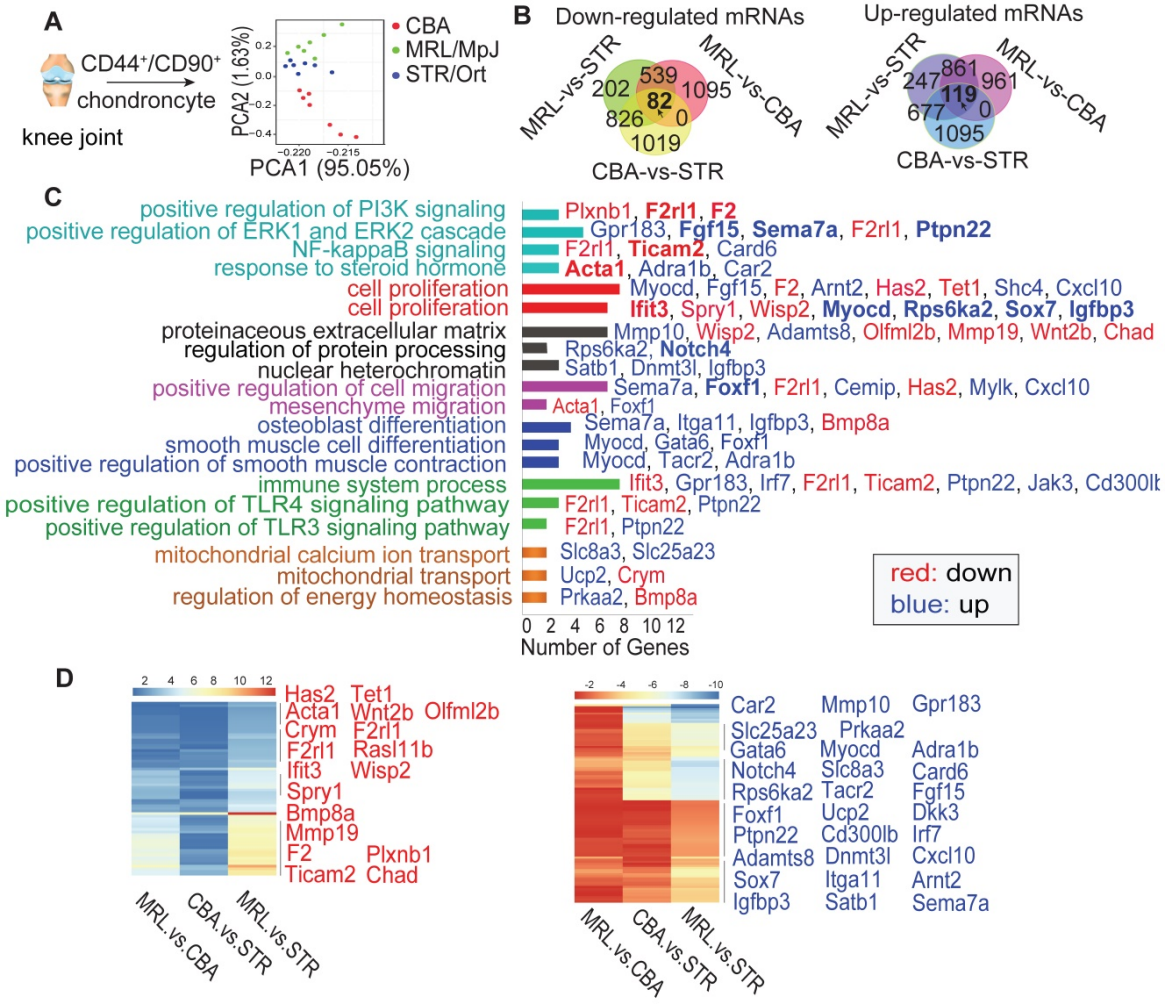
** RNA-sequencing results of CSPCs from STR/Ort, CBA and MRL/MpJ mice.** (**A**) Principal component analysis (PCA) from single-cell sequencing of CSPCs (n = 7) isolated from the joint cartilage of STR/Ort, CBA and MRL/MpJ mice at 5-month age. Venn diagram (**B**), KEGG (**C**) and Heatmap (**D**) analyses of the key molecules and signaling pathways differentially regulated in CSPCs from three mouse strains based on transcriptome analysis.

The CSPC are essential to maintain cartilage integrity by regulating chondrogenesis and matrix production 12. To understand the reason of RSK-3 enhancing cartilage repair, we made a single cell RNA sequencing of CSPC from MRL/MpJ, STR/Ort and CBA mice at 5-month age. Firstly, we collected CD44^+^ CD90^+^ chondrocytes (Figure s3A,B) and identified them as CSPC with positive markers (CD44, CD90, CD29 and Sca-1), and osteogenic, chondrogenic and adipogenic differentiation capacities (Figure s3C,D). RNA-sequencing results showed that the seven cells from each mouse strain showed similar tendency of gene expression, suggesting their representativeness (Figure s4). The cells in each strain had a unique RNA profile (Figure 2A), indicative of intrinsic differences at the cellular level. Venn analysis (Figure 2B) revealed that the intersections of the differentially expressed (DE) RNAs between MRL/MpJ versus CBA, MRL/MpJ versus STR/Ort, and CBA versus STR/Ort were associated with enhanced regenerative capacity. Specifically, the downregulated and upregulated mRNAs (Figure s5,s6) could be grouped according to various cellular functional and signaling pathways (Figure 2C,D and Figure s7), namely cell proliferation, migration and differentiation, PI3K signaling and the ERK cascade.

**Figure 3:**
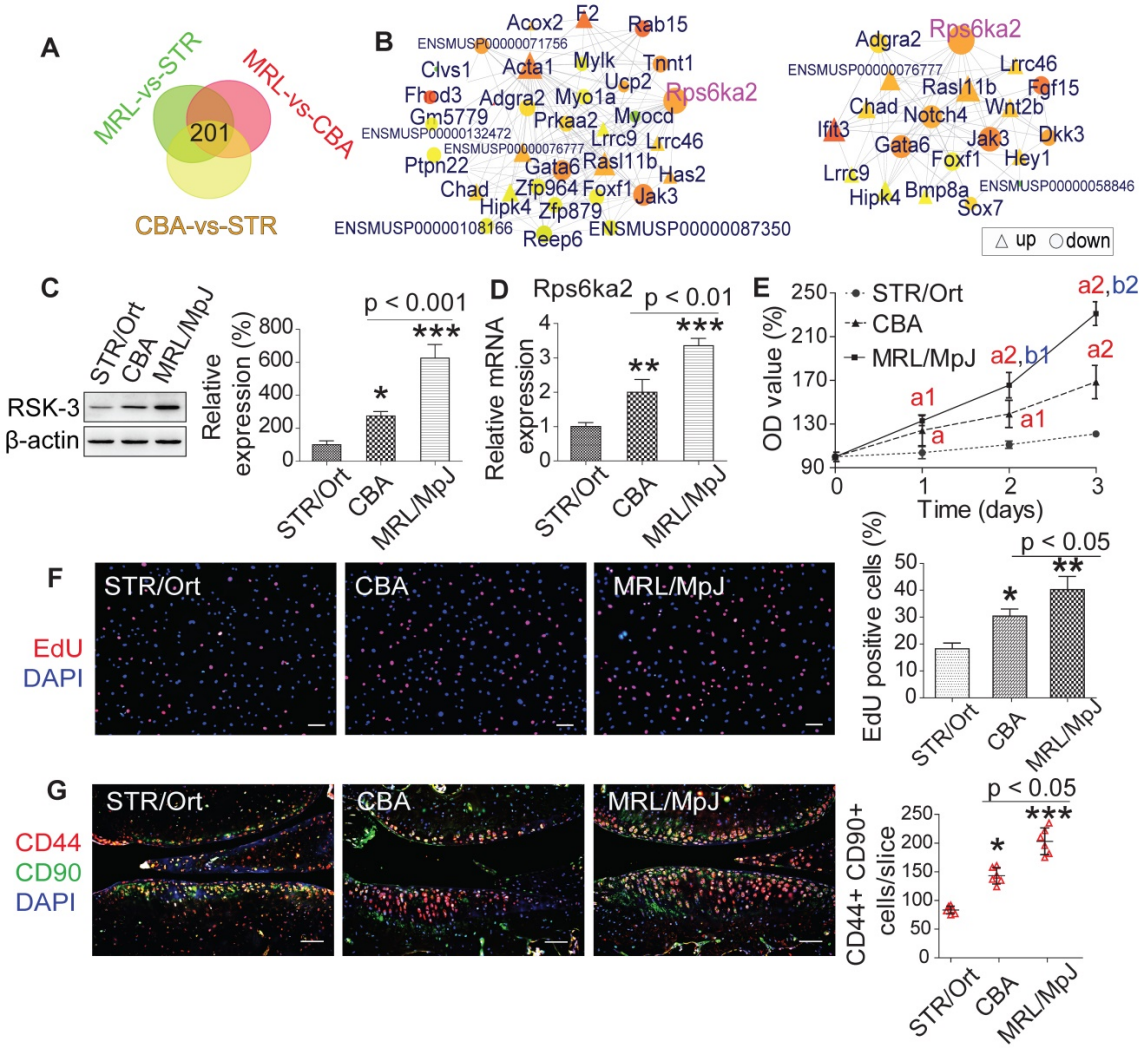
** RSK-3 contributes to different CSPCs proliferation ability in STR/Ort, CBA and MRL/MpJ mice.** (**A,B**) Venn and Regulatory network analysis confirms the key role RSK-3 in regulating CSPCs. (**C,D**) Expression of *Rps6ka2* and its encoding protein RSK-3 in CSPCs isolated from STR/Ort, CBA and MRL/MpJ mice at 5-month age. (**E,F**) Proliferative ability of CSPCs was monitored 0-3 days after culturing by CCK-8 and EdU assays. ^a^*p* < 0.05 versus STR/Ort; ^a1^*p* < 0.01 versus STR/Ort;^ a2^*p* < 0.001 versus STR/Ort; ^b1^*p* < 0.01 versus CBA;^ b2^*p* < 0.01 versus CBA (n = 8 per group). *p < 0.05, **p < 0.01 versus STR/Ort (n = 8 per group). (**G**) CD44^+^ CD90^+^ CSPCs were detected in the articular cartilage of STR/Ort, CBA and MRL/MpJ mice at 5-month age. *p < 0.05, ***p < 0.001 versus STR/Ort (n = 6 per group). Scale bars = 100 µm.

We subsequently constructed a network based on these DE RNAs (Figure s8). The core RNAs in this network, including *Rps6ka2* (encoding RSK-3), *Acta1*, *Ifit3* and *F2*, were predominantly associated with cell proliferation (Figure 2C). Finally, we validated the expression levels of a subset of these proliferation-related DE RNAs, namely *Rps6ka2*, *Sema7a*, *Igfbp3* and *Myocd*, and showed their down-regulated levels in human CSPC isolated from OA patients in comparison with those from non-OA samples (Figure s9). These data support that the proliferative capacity of CSPC may have a key role in cartilage repair. From the DE RNAs identified from the regulatory networks, *Rps6ka2* may be a key node with more connection to other nodes (Figure 3A,B). To validate our RNA-sequencing data, we determined the *Rps6ka2* gene and RSK-3 protein expression in CSPC from the CBA, STR/Ort and MRL/MpJ mice. Indeed, we found that RSK-3 was upregulated in CSPC from the CBA (3-fold gene and 1.4-fold protein increase) and MRL/MpJ (6-fold gene and 2-fold protein increase) mice compared with those from STR/Ort mice (Figure 3C,D).

**Figure 4:**
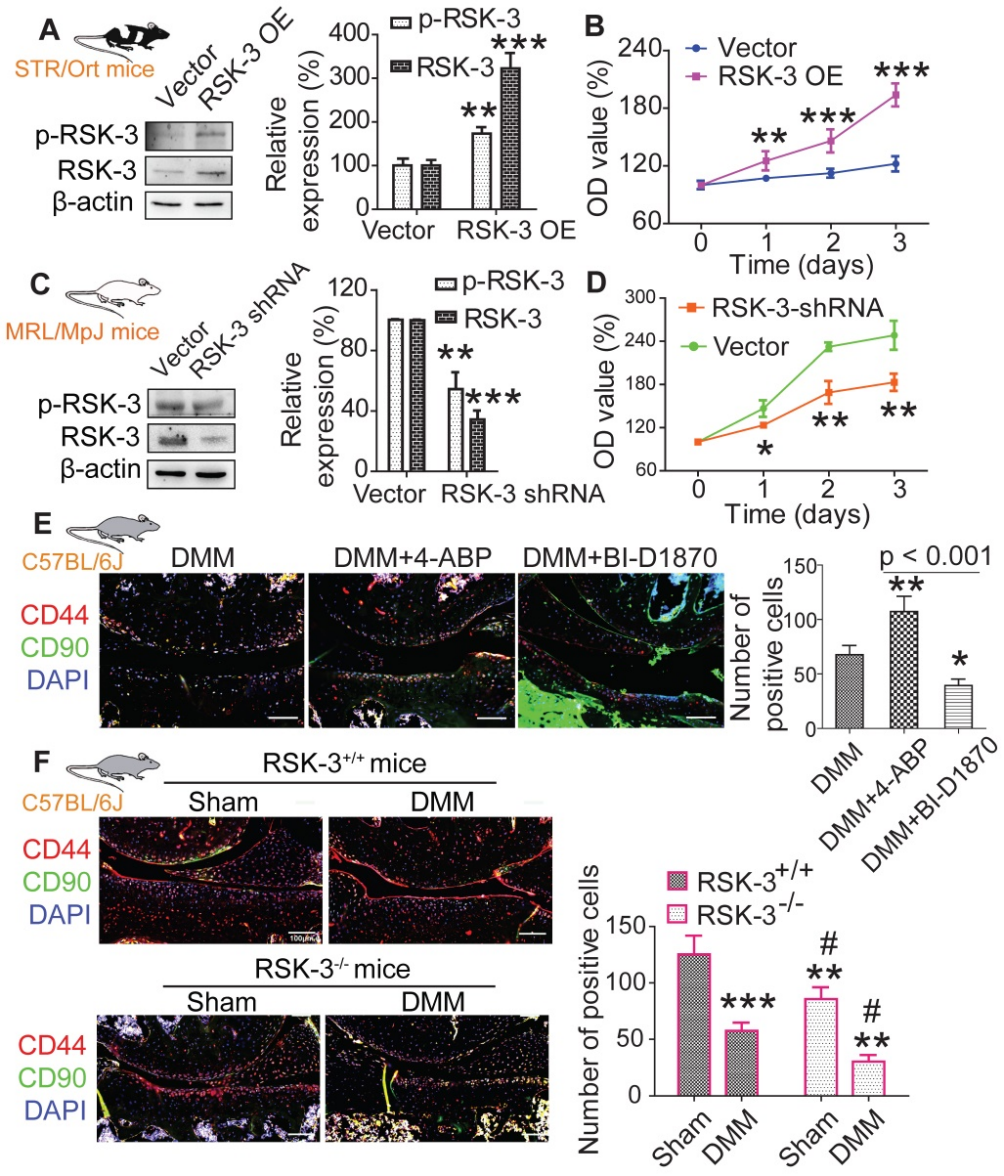
** RSK-3 promotes CSPCs proliferation.** (**A,B**) Overexpressed (OE) RSK-3 in STR/Ort CSPCs, and its effects on cell proliferation compared with empty vector, as assessed by CCK-8 assay. (**C,D**) short hairpin (sh) RNA-mediated RSK-3 downregulation in MRL/MpJ CSPCs, and its effects on cell proliferation, as assessed by CCK-8 method. *p < 0.05, **p < 0.01, ***p < 0.001 versus vector (n = 6 per group). (**E**) CD44 and CD90 labeling in knee joints of C57/BL6J mice subjected to DMM injury and 4-aminobiphenyl (4-ABP) or BI-D1870 for 4 weeks. *p < 0.05, **p < 0.01 versus DMM (n = 6 per group). (**F**) Immunolabeling of CD44 and CD90 in articular cartilage from RSK-3^+/+^ and RSK-3^-/-^ mice subjected to DMM injury or sham procedure. **p < 0.01, ***p < 0.001 versus sham (RSK-3^+/+^), ^#^*p* < 0.05 versus DMM (RSK-3^+/+^) (n = 6 per group). Scale bars = 1000 µm.

After sorting CSPCs with CD44 and CD90 positive markers using flow cytometry, we analyzed the proliferative potential of cultured CSPC (passage 3) from MRL/MpJ, CBA and STR/Ort mice. CCK-8 and EdU assays showed that the proliferation rate of CSPC from MRL/MpJ (40%) exhibited a significant increase versus CBA (30%) and STR/Ort (19%) mice (Figure 3E,F). Additionally, the number of CSPC in STR/Ort murine cartilage (80 cells) was markedly lower than that in the other two strains, with the highest number of cells present in MRL/MpJ cartilage (186 cells) (Figure 3G). To further confirm the function of RSK-3 in CSPC proliferation, we examined the effects of upregulating and downregulating the expression of RSK-3 on CSPC self-renewal. The overexpression of RSK-3 in CSPC from the STR/Ort mice significantly increased cell proliferation (Figure 4A,B). By contrast, RSK-3 knockdown or inhibition markedly restrained cell growth in the MRL/MpJ mice (Figure 4C,D). Up-regulated RSK-3 increased the number of CSPC in cartilage, while inhibiting RSK-3 expression suppressed CSPC growth (Figure 4E). Furthermore, the RSK-3^-/-^ mice showed a reduced number of CSPC in the cartilage tissue compared with wild-type (C57BL/6) OA mice (Figure 4F). Altogether, these data suggest that RSK-3 is a key regulator of CSPC proliferation and cartilage repair in OA.

### rpS6 and autophagy are activated by RSK-3

**Figure 5:**
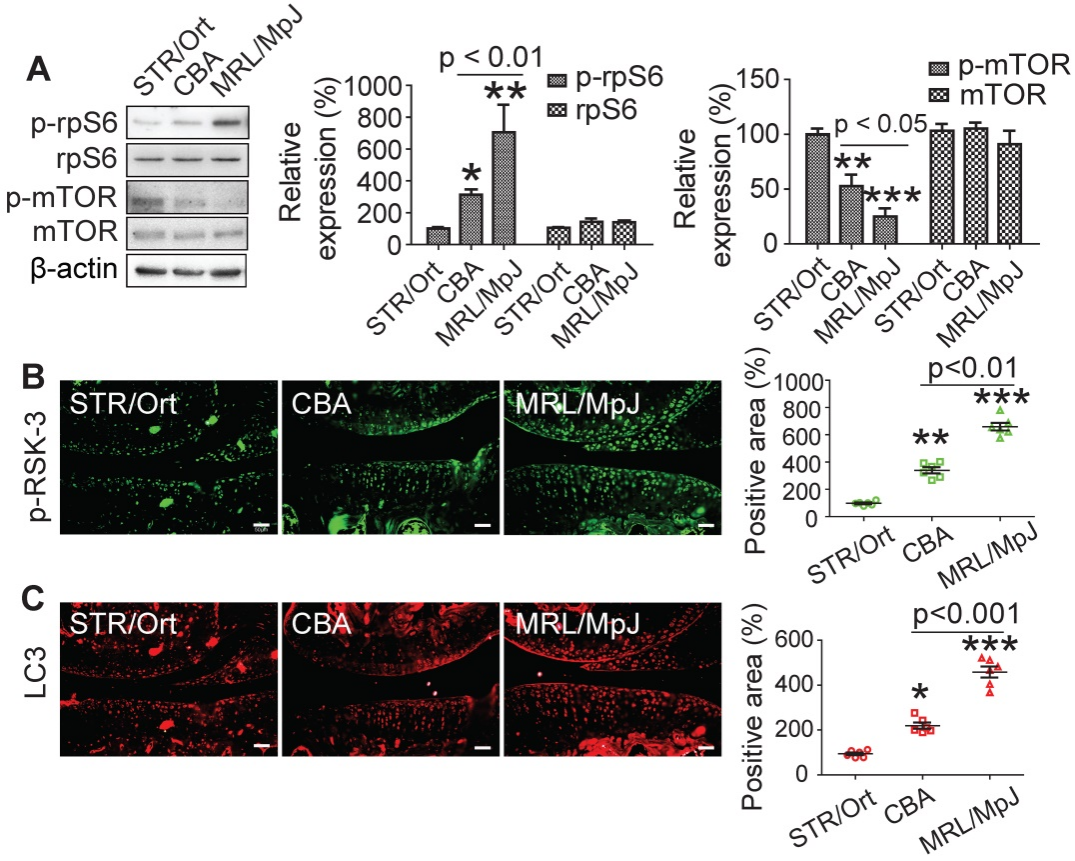
** CSPCs from MRL/MpJ mice show high expression levels of rpS6 and autophagy.** (**A**) Protein expression of p-rpS6, rpS6, p-mTOR and mTOR in CSPCs from STR/Ort, CBA and MRL/MpJ mouse. Immunolabeling of p-RSK-3 (**B**) and LC3 (**C**) in cartilage isolated from each mouse strain. Scale bars = 50 µm. *p < 0.05, **p < 0.01, ***p < 0.001 versus STR/Ort (n = 6 per group).

**Figure 6:**
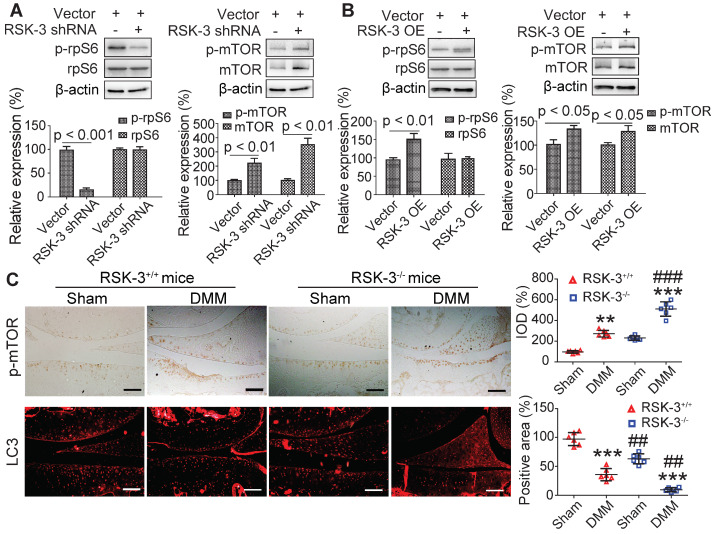
** RSK-3 promotes cell proliferation by regulating rpS6 and autophagy.** Effects of downregulated RSK-3 in MRL/MpJ CSPCs (A) and overexpressed (OE) RSK-3 in STR/Ort CSPCs (B) on the protein expression of p-rpS6, rpS6, p-mTOR and mTOR. *p < 0.05, **p < 0.01, ***p < 0.001 versus STR/Ort (n = 6 per group). (C) Immunolabeling of p-mTOR and LC3 in articular cartilage after DMM injury in normal and RSK-3^-/-^ mice. **p < 0.01, ***p < 0.001 versus sham (RSK-3^+/+^), ^#^*p* < 0.05, ^###^*p* < 0.001 versus DMM (RSK-3^+/+^) (n = 6 per group). Scale bars = 100 µm.

rpS6 and mTOR are the major down-stream molecules of RSK-3 25. Decreasing rpS6 expression with specific shRNA inhibited the proliferative capacity of CSPC from the CBA mice (Figure s10), confirming the pivotal role of rpS6 in modulating CSPC growth. We also observed that CSPC with high proliferative ability have increased levels of p-rpS6 (Figure 5A). Downregulating the expression of RSK-3 by specific shRNA significantly reduced the level of p-rpS6 (Figure 6A), whereas the overexpression of RSK-3 caused an increased level of p-rpS6 in the CSPC (Figure 6B). Therefore, the RSK-3/rpS6 pathway may be implicated in the regulation of the CSPC proliferation.

The capacity of the CSPC growth was opposite to the level of p-mTOR (Figure 5A). mTOR is normally considered to serve as a crucial role in regulating autophagy 33. As an initial step to unveil the relationship between RSK-3 and mTOR-mediated autophagy, we detected the expression of RSK-3 and LC3 in cartilage of STR/Ort, CBA and MRL/MpJ mice. Following the increased expression of p-RSK-3 (Figure 5B), a correlated increase in the expression of LC3 in the cartilage was observed, which is indicative of the possible involvement of autophagy (Figure 5C). However, it was shown that either of the downregulation or upregulation of the RSK-3 expression could elevate the level of p-mTOR (Figure 6A,B), and the DMM injury induced high levels of p-mTOR in the cartilage, which were further amplified in RSK-3^-/-^ mice (Figure 6C). These results suggest that mTOR may not be solely regulated by RSK-3 though the autophagy inhibition was observed in degenerative or impaired cartilage of mice.

### mTOR induced autophagy is not involved in RSK-3 mediated cell proliferation

**Figure 7:**
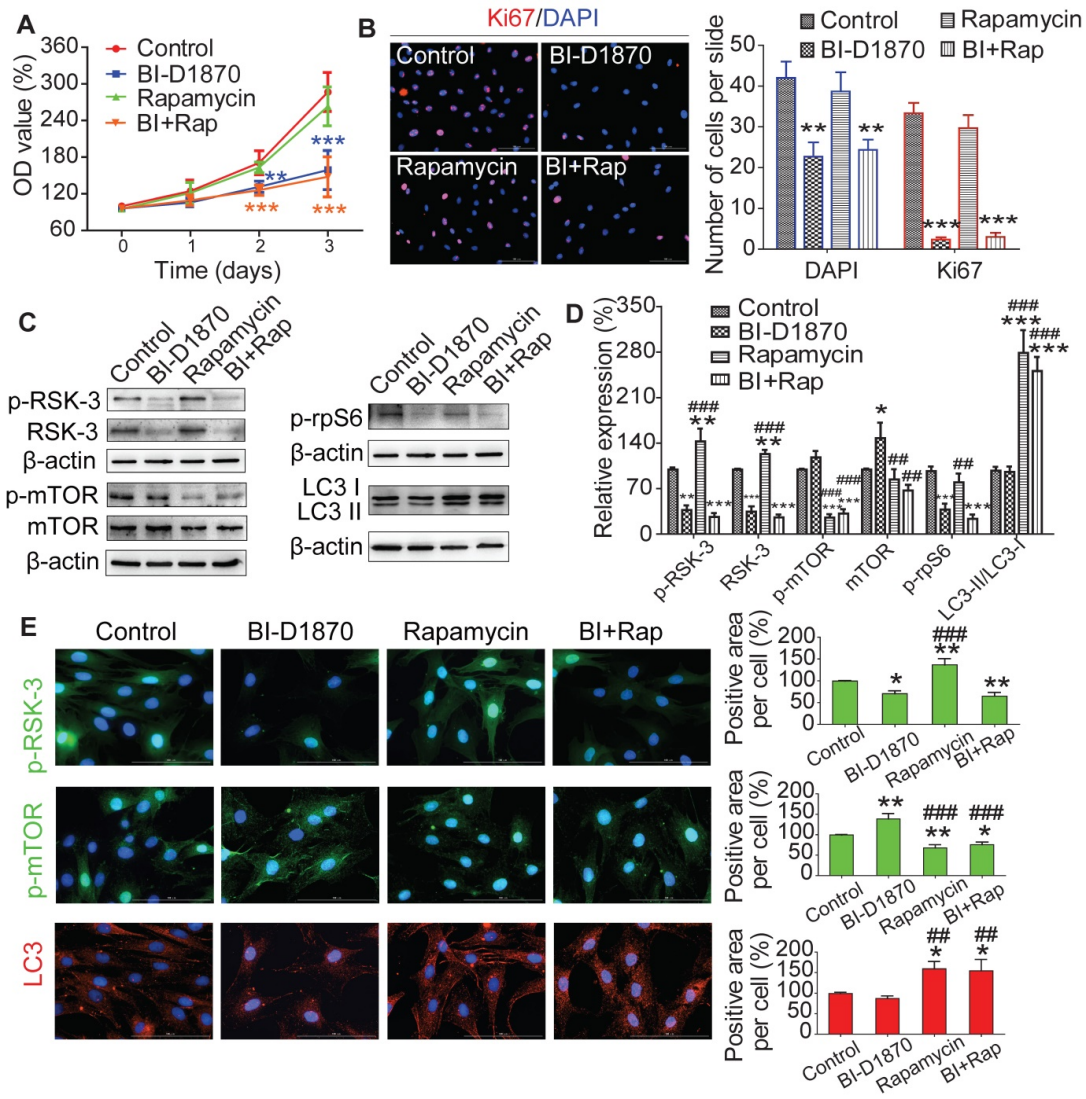
** RSK-3 activates rpS6, but not autophagy, to regulate CSPCs proliferation.** The proliferative ability of CSPCs from C57/BL6J mice after RSK-3 inhibitor (BI-D1870) and mTOR inhibitor (rapamycin) treatment detected by CCK-8 assay (**A**) and Ki67 labeling (**B**). (**C,D**) Protein expression of RSK-3, p-RSK-3, mTOR, p-mTOR, p-rpS6 and rpS6 in CSPCs from C57/BL6J mice and the corresponding quantifications. (**E**) p-RSK-3, p-mTOR and LC3 immunolabeling in CSPCs from C57/BL6J mice. *p < 0.05, **p < 0.01, ***p < 0.001 versus control, ^##^*p* < 0.01, ^###^*p* < 0.001 versus BI-D1870 (n = 6 per group). Scale bars = 100 µm.

To understand the role of RSK-3/mTOR in modulating cell proliferation, we treated CSPC from C57BL/6J mice with an RSK-3 inhibitor (BI-D1870) or an mTOR inhibitor (rapamycin). BI-D1870, but not rapamycin, significantly inhibited cell proliferation (Figure 7A,B). Furthermore, the inhibition of RSK-3 significantly decreased the expression level of p-rpS6 and partially elevated the level of p-mTOR (Figure 7C-E). As expected, rapamycin markedly inhibited the expression of p-mTOR and induced autophagy, but also increased the level of RSK-3 and p-RSK-3 (Figure 7C-E). However, in the RSK-3-shRNA treated cells, rapamycin induced autophagy but did not promote cell proliferation (Figure s11). Overall, these data suggested that mTOR-mediated autophagy did not promote CSPC proliferation and the RSK-3/rpS6 pathway may there be the most important regulator on cell proliferation.

## Discussion

This study aimed to unveil the role of RSK-3 and the potent mechanism in enhancing cartilage repair. Our data herein provide several important findings: (1) the proliferative ability of endogenous CSPC is critical for cartilage repair; (2) RSK-3 loss aggravates cartilage lesions in OA mice; (3) the upregulation of RSK-3 promotes CSPC proliferation; (4) RSK-3 is essential for cartilage repair. Specifically, these data identify RSK-3 as a key modulator of CSPC and therefore a potential molecular target on which to develop novel therapeutic strategies.

The STR/1N strain is obtained during tumor-resistance selection with carcinogens 34, and STR/Ort mice are derived from the STR/1N strain following a period of non-inbreeding 19. It is reported that chromosome 4 and 8 are the potential OA associated locus in STR/ort mice 35,36. The detailed genetic information of STR/ort mice is still not clear. CBA mice are originally created in inbred strain by crossing a Bagg albino female with a DBA/2 male (https://www.jax.org/strain/000656) 37. The CBA mouse lack of overt OA are the parental strain of STR/Ort mice 16, 17, 19, 20, and widely used as the control of STR/Ort mice. The MRL/MpJ strain is generated from multiple strains including C57BL/6J (0.3%), C3H/HeDi (12.1%), AKR/J (12.6%) and LG/J (75%) and then maintained by inbreeding (https://www.jax.org/strain/000486). MRL/MpJ mice, as the super healer mouse, can heal the injury of articular cartilage by some genes, such as *Wnt16, Axin2, Xrcc2 and Pcna*. LG/J mice also exhibit similar, but not superior, articular cartilage and ear wound healing capability 38. The genetic backgrounds of the three strains and their current gene modulation information may provide important clues for further studies to understand the regenerative mechanism.

Although STR/Ort mice can spontaneously develop cartilage degeneration of the joint 12,16, we used the DMM-induced OA model to further accelerate the progression of cartilage damage and to evaluate the regenerative effects across three different murine strains. Consistent with previous reports 22,39, we found that the injured joints of the STR/Ort, CBA and MRL/MpJ mice exhibited different cartilage repair capacities.

Our previous study showed that fibronectin-selected CSPC from STR/Ort mice had limited proliferative and chondrogenic differentiation potential, in line with the low potential of endogenous cartilage repair 12. Here, to further characterize the key regulator of cartilage repair, we used primary CSPC freshly sorted form articular cartilage for transcriptional analysis, which eliminated the effects of cell heterogeneity resulted from cell culture conditions. It was noted that we were unable obtain a large number of CSPC, due to the small samples of mouse cartilage, long time (1 h) for carriage isolation of each mice, hard digestion to single cell with multiple enzymes, and low proportion of CSPC in cartilage. Therefore, we were only able to collect seven cells from each murine species for single-cell RNA sequencing. Despite this limitation, the PCA analysis clearly generated three clusters from the three strains (Figure 2A) and the gene expression in the seven cells from each strain had relative consistent tendency (Figure s4), suggesting that these seven cells per mouse strain were representative of the stem cell population. Consistent with previous microarray and RNA-sequencing studies 20,40, our results revealed that the nuclear factor (NF)-κB signaling pathway, osteogenic differentiation and inflammation were associated with OA in STR/Ort mice. Most notably, we identified that many molecules, especially the core ones (such as *Rps6ka2, F2, Ifit3*) in the regulatory networks, were involved in the positive and negative regulation of cell proliferation.

RNA-sequencing revealed that RSK-3 was a key molecule underlying CSPC proliferation. Notably, the expression of RSK-3 was correlated with regenerative capacity, such that high expression levels of RSK-3 were found in MRL/MpJ mice that were not susceptible to OA, whereas low expression levels were found in STR/ort and CBA mice that were highly and moderately susceptible to OA, respectively. RSK-3 belongs to a family of serine/threonine kinases and consists of four human isoforms (RSK1-4) that regulate diverse cellular processes, including cell growth, motility, survival and proliferation 25. The RSK isoforms are directly activated by ERK1/2 or PDK1 via the Ras-ERK/MAPK signaling cascade upon growth factor, hormone, neurotransmitter or chemokine stimulation 25. Notably, RSK-3 has previously been implicated in cell-cycle progression 41.

It has been shown that RSK-mediated Raptor phosphorylation is required for mTORC1 activation by the Ras/MAPK pathway, which is important for cell growth 42. The phosphorylation of rpS6 via RSK is not exclusively independent of the mTORC1 pathway as mTOR is regulated by multiple molecules including AKT 25,43. Our study also showed that the RSK-3 shRNA-mediated inhibition of cell proliferation was companied with the AKT activation and elevated p-AKT expression (Figure s12). Therefore, it may be speculated that AKT activates mTOR when RSK-3 is inhibited and serves as a potential compensatory mechanism to maintain the CSPC proliferative capacity. These results anyhow suggest the potential role of the AKT/mTOR/rpS6 pathway on the proliferation of CSPC though more detailed studies are still ongoing to further decipher the mechanisms underlying the RSK-3-related or independent of the rpS6 regulation. In this respect, we in this study showed that a low expression of RSK-3 did not sufficiently activate rpS6, despite a potential compensatory activation of mTOR by AKT, and thus inhibited CSPC proliferation. Thus, the RSK-3/rpS6 pathway seemed to play a critical role in promoting CSPC proliferation. In this study, we just made a preliminary explanation about the RSK-3-independent regulation on rpS6, which still requires further studies by us and others.

Several mechanisms include cell cycle regulation and DNA repair, Wnt pathway, and dampened inflammatory response, appear to promote articular cartilage healing in MRL/MpJ mice 44. Moreover, the accelerated cartilage repair in the DMM cartilage injury model is related with the down-regulation of Wnt/β-catenin and TGF-β/pSMAD 2/3 signaling, up-regulation of BMP/pSMAD5 signaling, and lower levels of ADAMTS4, MMP9, and MMP13 in MRL/MpJ mice 22. In STR/Ort mice, multiple factors affecting matrix remodeling, chondrocyte metabolism, oxidative stress, immune response and metabolic system are possibly involved in the spontaneous OA progression 16. These studies mainly focused on the cartilage or chondrocytes, and provided only the overall information but not sufficiently specified. By analyzing the difference between CSPC isolated from the STR/Ort and MRL/MpJ mice, respectively, the RSK-3/rpS6 pathway is shown to be involved in regulating the CSPC proliferation, which may be a key mechanism underlying the variable regenerative competences as observed in the two distinct mouse strains.

Various important signaling pathways that have been experimentally shown to regulate CSPC and cartilage healing include TGF-β, Wnt/β-catenin, CBFβ-Runx1 and EGFR 45. Additionally, BMPs, FGFs, HIF, NF-κB, MAPK and hedgehog cascades are also involved in modulating cartilage regeneration and degradation 46. So a complex network could be speculated for the CSPC regulation. In this study, we just validated the key role of RSK-3 on the CSPC proliferation. Many other molecules, such as Bmp8a, Itga11, Myocd, Acta1 and Adamts8 47-51, are also known to be amongst the key regulators involved in matrix synthesis and degradation, migration, differentiation, inflammatory modulation and energy homeostasis (Figure 2). All of these may contribute to CSPC-mediated OA mechanisms. Therefore, further work is required to more deeply and extensively understand the function of key molecules and the regulatory networks in cartilage homeostasis and regeneration, which may in turn sever as therapeutic targets of OA.

## Conclusion

Our results demonstrate that RSK-3 contributes to the cartilage repair at least in part by promoting CSPC proliferation. Specific activation of RSK-3 may therefore offer therapeutic potential for OA or related joint diseases.

## Supplementary Material

Supplementary figures and tables.

## Abbreviations

CSPC: cartilage stem/progenitor cells
OA: osteoarthritis
RSK-3: Ribosomal s6 kinase 3
rpS6: ribosomal protein S6
MMPs: matrix metallopeptidases
TIMPs: tissue inhibitors of metalloproteinases
MAPK: mitogen-activated protein kinase
JNK: c-Jun N-terminal kinase
ERK1/2: extracellular regulated kinase 1/2
FGF2: basic fibroblast growth factor
4-ABP: 4-aminobiphenyl
DMM: destabilization of the medial meniscus
RSK3^-/-^: RSK-3 knockout
OARSI: Osteoarthritis Research Society International
CCK-8: Cell Counting Kit-8
dsOligo: double-stranded oligonucleotides
BSA: bovine serum albumin
ANOVA: one-way analysis of variance
DE: differentially expressed
