# Supplementary material for: RSK-3 promotes cartilage regeneration via interacting with rpS6 in cartilage stem/progenitor cells
Source: Theranostics. 2020 May 25;10(15):6915–27. doi: 10.7150/thno.44875 (PMC7295041; doi:10.7150/thno.44875)
Supplement: Supplementary file 1 — Supplementary figures and tables. [file thnov10p6915s1.pdf]

Supplemental methods

Flow cytometry

To confirm the stem cell characters of $CD44^+$ $CD90^+$ cells, the culture-expanded cells (passage 1, $1x10^6$) were harvested by trypsin digestion, blocked with 2% FBS at 4˚C for 30 min and then incubated individually with FITC-conjugated CD44, Sca-1, CD34, CD45 or PE-conjugated CD90, CD29 antibodies (BD Biosciences, USA) at 4˚C for 30 min. Unlabeled cells were used as a control for all antibodies. The cells were resuspended in 0.4 ml phosphate-buffered saline (PBS) and analyzed on a FACSCalibur (BD Biosciences, USA). Data were processed using FlowJo7.6 software (Java Software).

Multilineage differentiation

The osteogenic, chondrogenic and adipogenic differentiative capacity of $CD44^+$ $CD90^+$ cells (passage 1) were detedted according to our previous methos.[1] Briefly, the cells were cultured in a 24-well culture plate (1×10^4 cells per well), and indued in osteogenic differenciation medium (100 nM dexamethasone, 10 mM β-glycerol phosphate and 50 μM ascorbate-2-phosphate in high-glucose DMEM with 10% FBS) for 28 days, chondrogenic differentiation medium (100 nM dexamethasone, 1% insulintransferrin-sodium selenite, 50 μM ascorbate-2-phosphate, 1 mM sodium pyruvate, 50 ng/ml TGF-β1 in high-glucose DMEM with 10% FBS) for 21 days, and adipogenic differentiation medium (100 nM dexamethasone, 10 μM insulin, 0.5 mM isobutyl-methylxan-thine and 200 μM indomethacin in high-glucose DMEM with 10% FBS) for 14 days. The success of differentiation were confirmed by staining the cells with Alizarin Red S for osteogenesis, toluidine blue for chondrogenesis and Oil-red O for adipogenesis.

References

1. Zhang S, An Q, Hu P, Wu X, Pan X, Peng W, et al. Core regulatory RNA molecules identified in articular cartilage stem/progenitor cells during osteoarthritis progression. Epigenomics. 2019; 11: 669-84.

Table s1 Primer sequences used for real-time PCR assay (human)

Primers	Sequence (5' to 3')
F2	CAGCTATGGAGGAGTCGCTAC
	CGGTGGGGTCTGTAGTGTAG
Myocd	CCTGCAGCTCCAAATCCTCAG
	GGGTATTGCTCAGTGGCGTTG
Ifit3	AGCCCAACAACCCAGAATTCTC
	CTCAGCTCAATGGCCTGCTTC
Igfbp3	GTGTCTGATCCCAAGTTCCAC
	GGACTCGGAGGAGAAGTTCTG
Sema7a	GAGGACGTGGCAAGGTCTAC
	CAGTCCCGCTTATCCAGACAG
Rps6ka2	ACGCTGTACCGGAAGGAGATC
	CTCGTAGCCATCGGTGAAGTG
β-actin	CATGTACGTTGCTATCCAGGC
	CTCCTTAATGTCACGCACGAT

Table s2 Primer sequences used for real-time PCR assay (mice)

Primers	Sequence (5' to 3')
Rps6ka2	CTCAGCAACGCCTAACAGCAG
	CCATGGCACCCTTCACTAGATG
β-actin	CATGTACGTTGCTATCCAGGC
	CTCCTTAATGTCACGCACGAT

Supplemental results

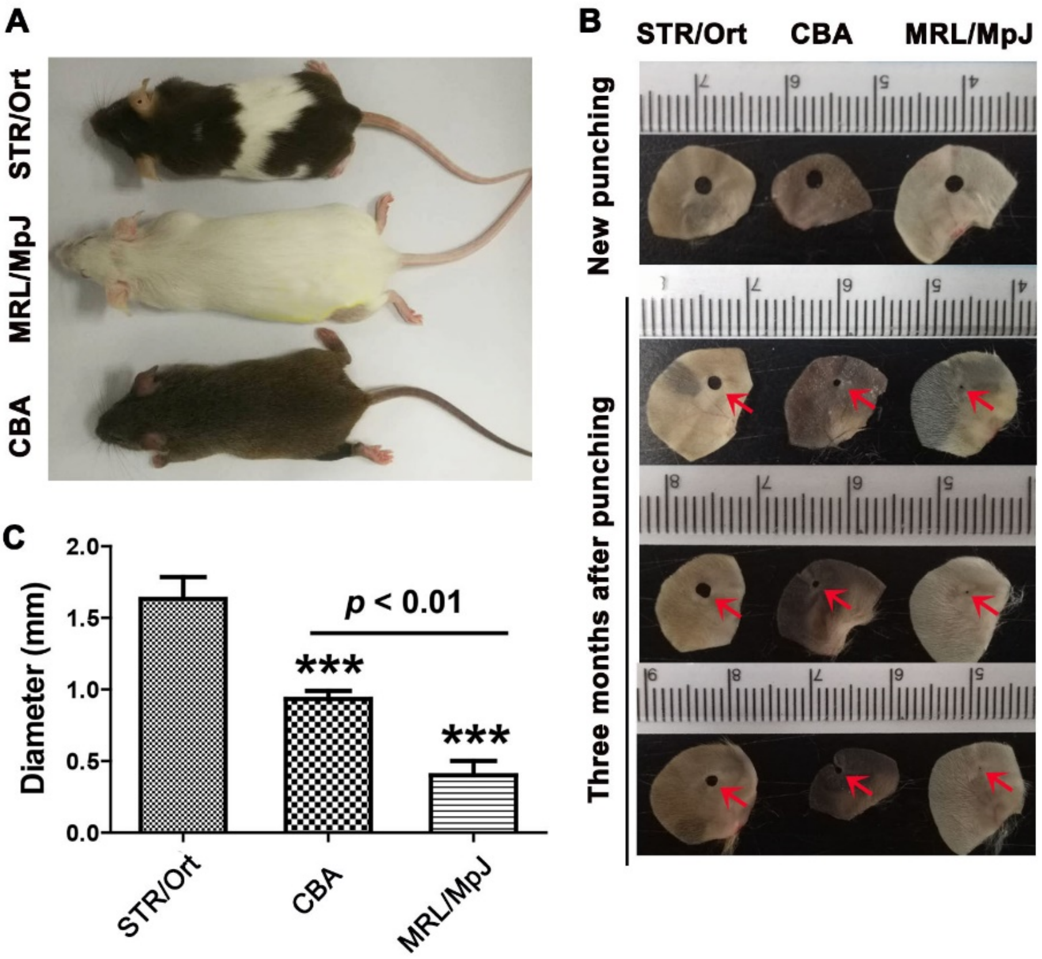

Figure s1 Ear-hole closure assay as an indicator of tissue regenerative capacity in three mouse strains. A: STR/Ort, CBA and MRL/MpJ mice; B-C: Changes in ear hole diameter, 3 months after inducing an initial 2-mm-diameter injury. ***$p < 0.001$ versus STR/Ort. n = 6 mice per group.

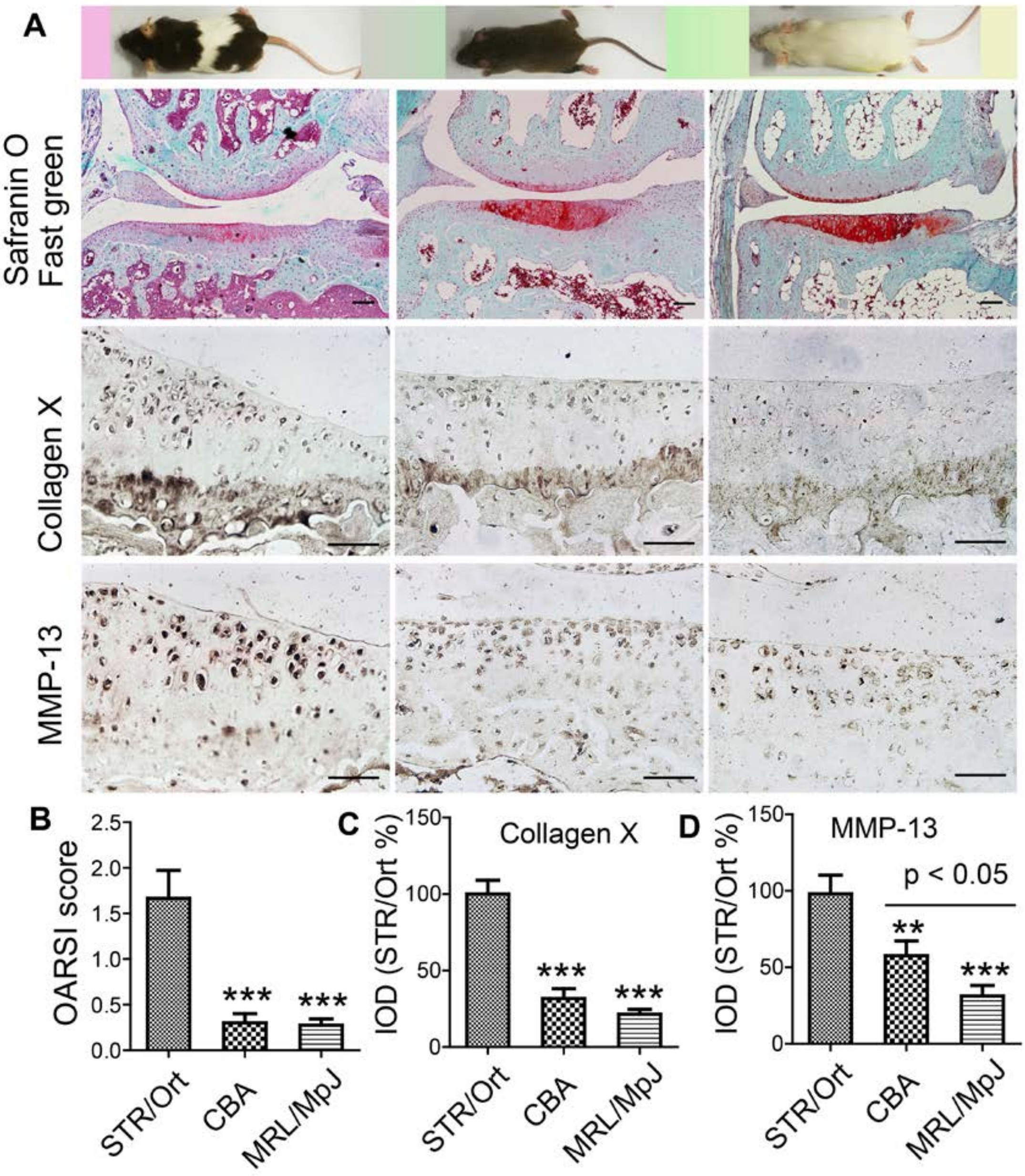

Figure s2 Cartilage degeneration in the knee joints of STR/Ort, CBA and MRL/MpJ mice at 8 months of age. (A) Mouse knee joints were stained with safranin-O/fast green, Collagen X and MMP-13. (B) OARSI scores for cartilage degeneration assessment. (C,D) Immunohistochemical analysis of image gray integral optical density (IOD). ***p* < 0.01, ****p* < 0.001 versus STR/Ort. n = 6 mice per group. Scale bars = 100 μm.

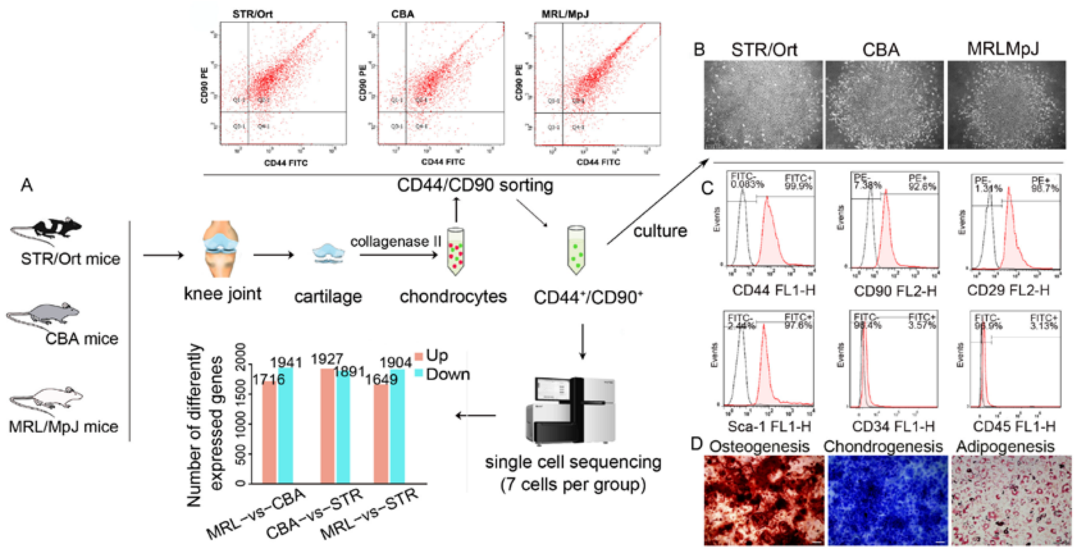

Figure s3 Single-cell RNA sequencing of CD44$^+$ CD90$^+$ CSPCs isolated from the joint cartilage of STR/Ort, CBA and MRL/MpJ mice. Isolated CD44$^+$ CD90$^+$ cells were divided into two groups for analysis by single-cell RNA sequencing (A) or culturing (B) and subsequent identification by flow cytometry (C) and multilineage differentiation including osteoblastic (alizarin red staining), chondrogenic (toluidine blue staining) and adipocytic (oil red staining) (D). n = 3, scale bar = 100 μm.

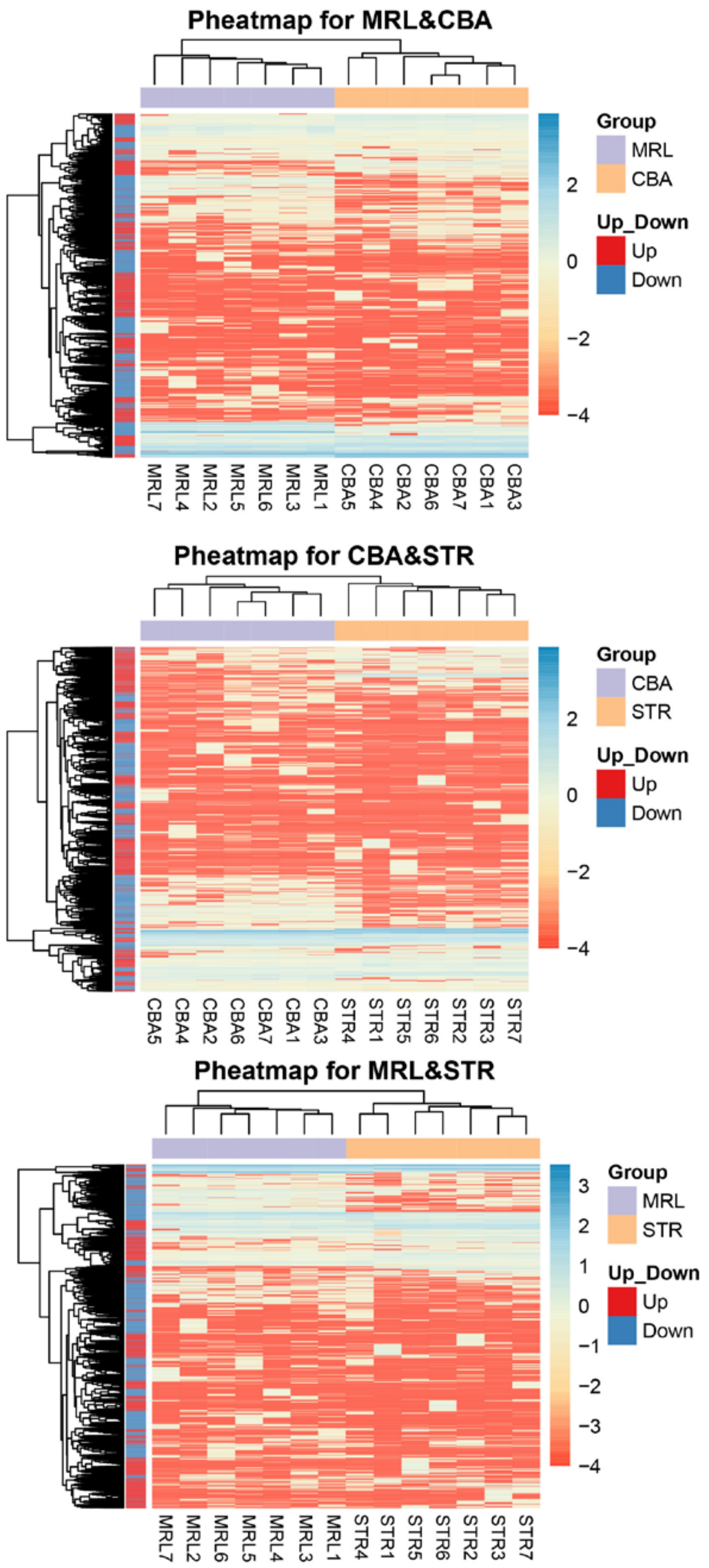

Figure s4 Heatmap of the gene expression in each cell from MRL/MpJ, CBA and STR/Ort mice.

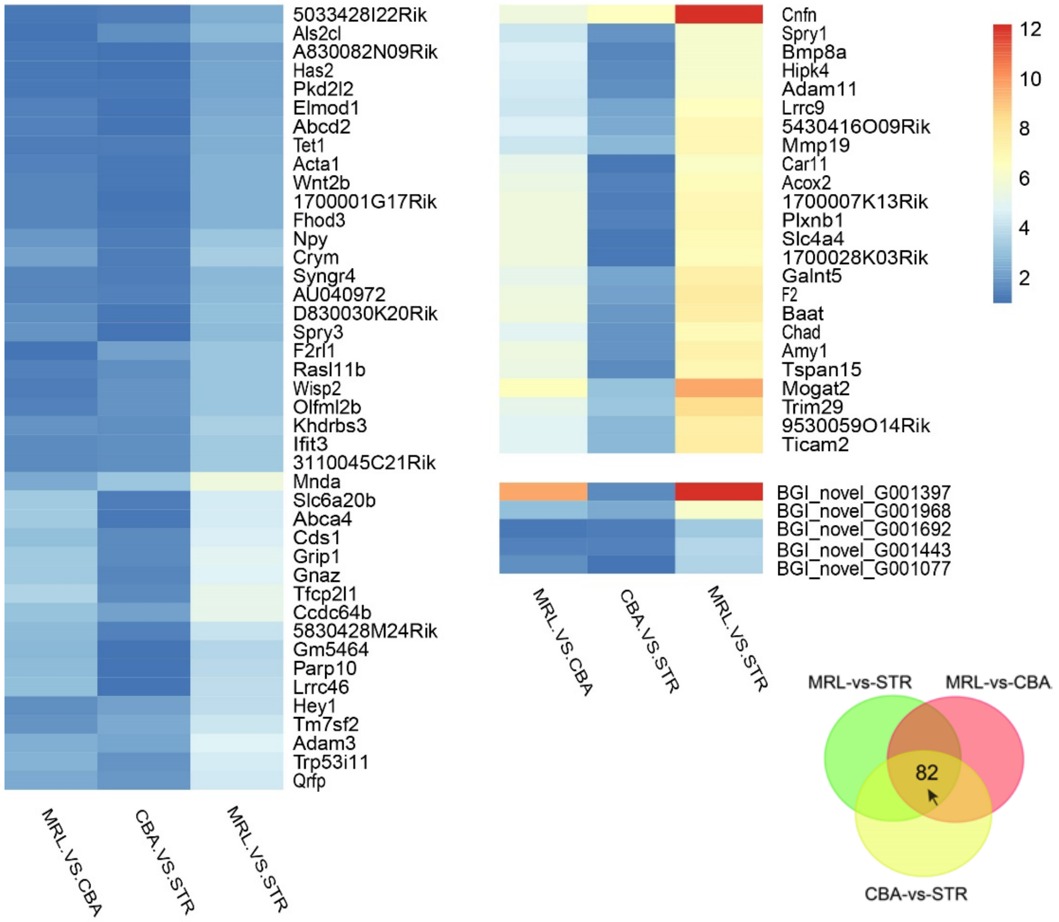

Figure s5 Heatmap of the differentially down-regulated RNAs in the intersection (black arrow) of the differentially expressed RNAs between MRL vs. CBA, CBA vs. STR and MRL vs. STR mice.

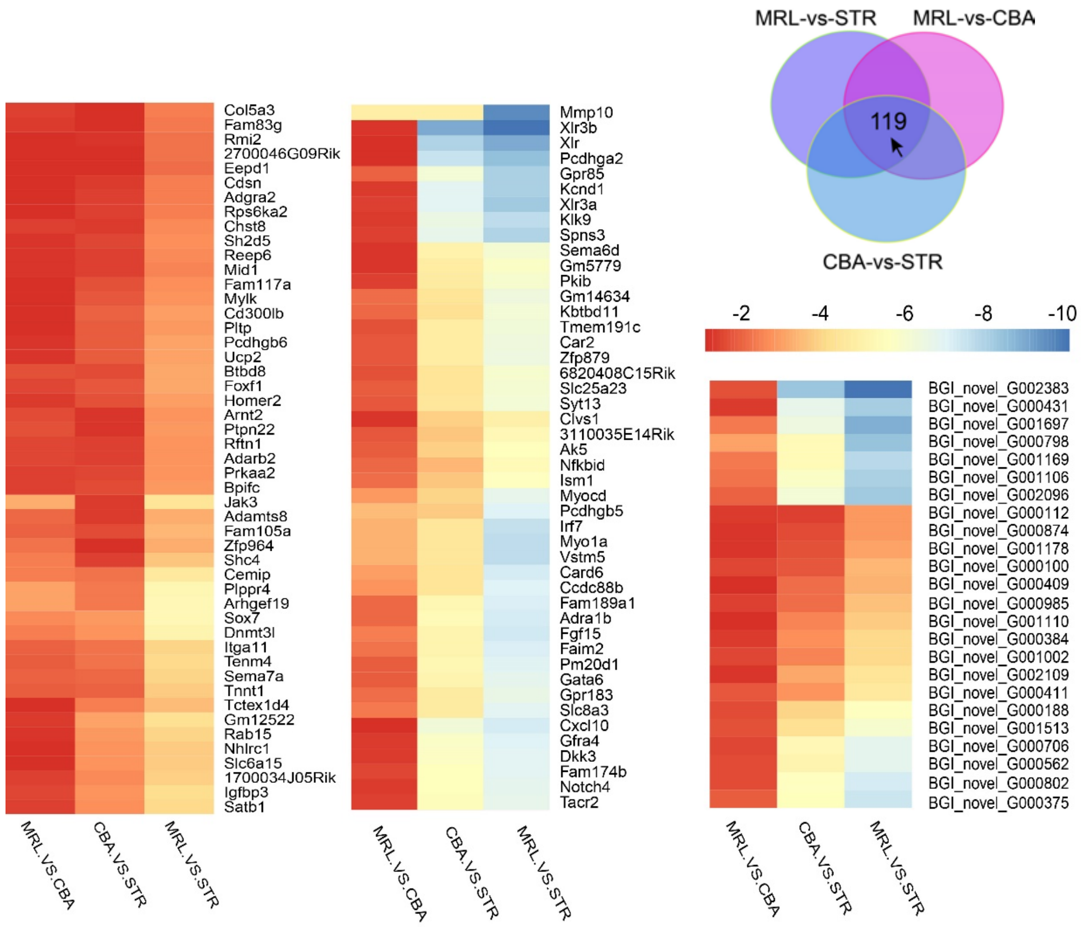

Figure s6 Heatmap of the differentially up-regulated RNAs in the intersection (black arrow) of the differentially expressed RNAs between MRL vs. CBA, CBA vs. STR and MRL vs. STR mice.

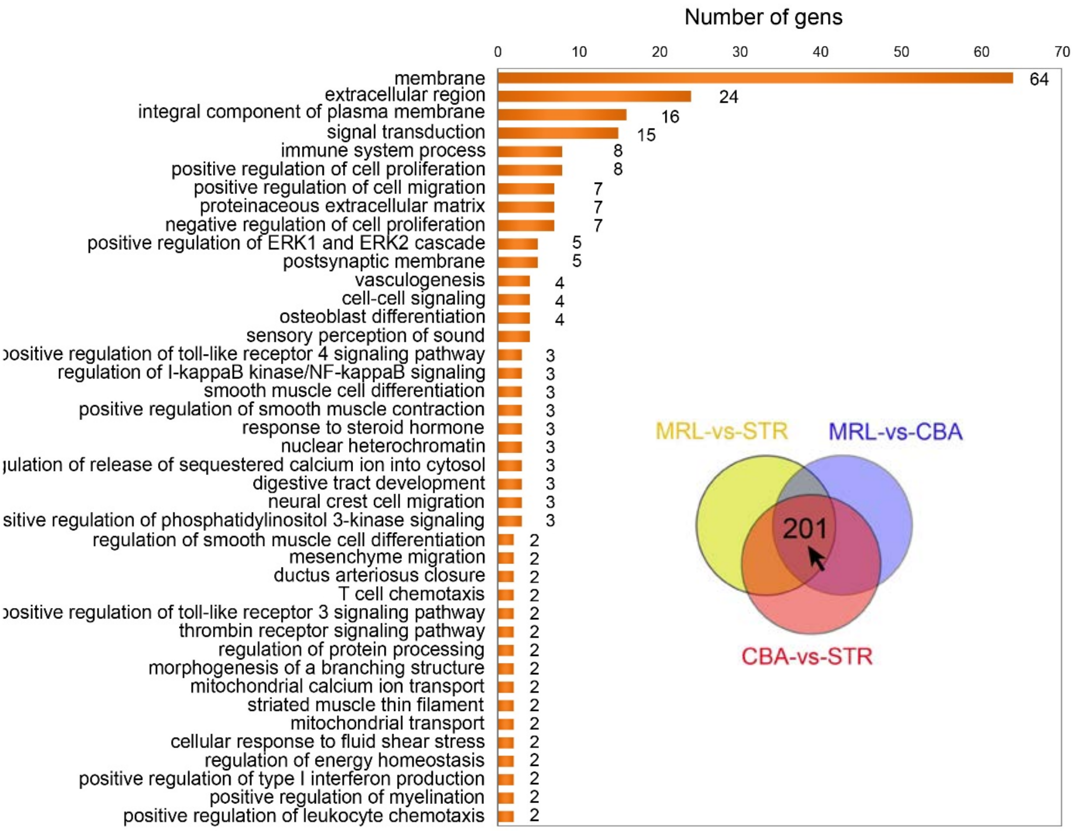

Figure s7 Pathway enrichment analysis of differentially expressed RNAs in the intersection (black arrow) of the differentially expressed RNAs between MRL vs. CBA, CBA vs. STR and MRL vs. STR mice.

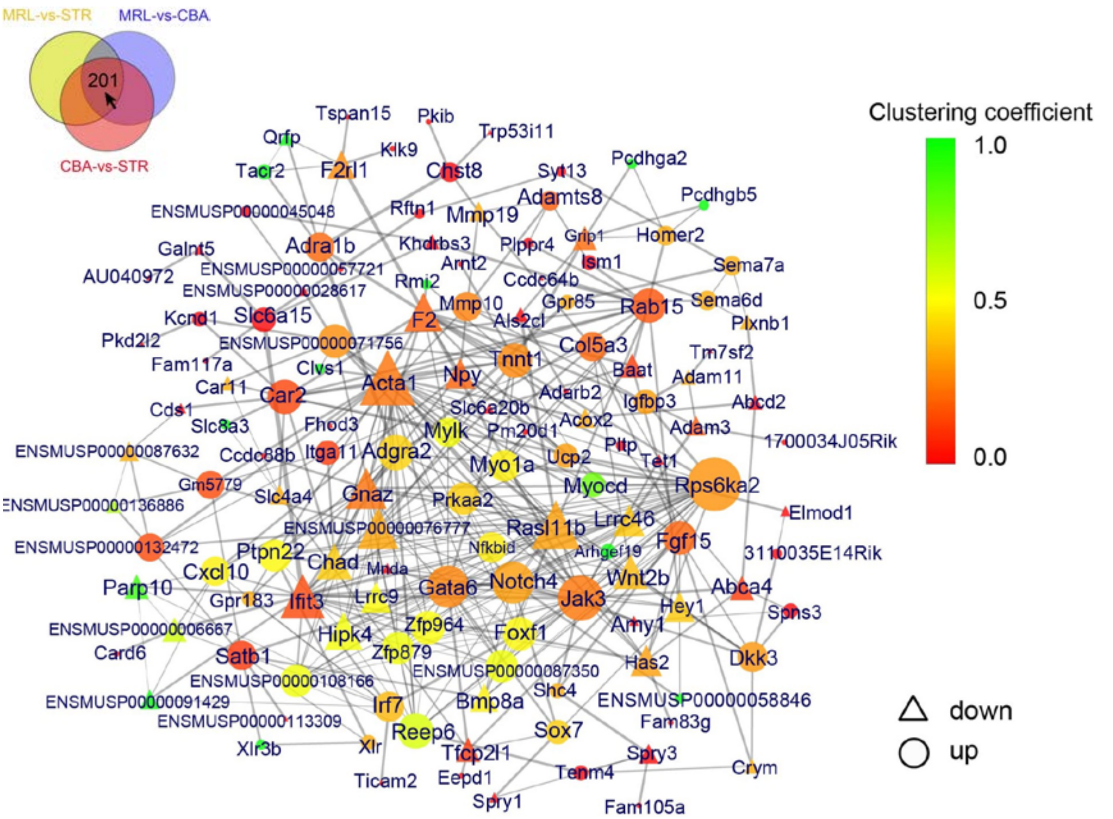

Figure s8 Regulatory network based on the differentially expressed RNAs in the intersection (black arrow) of the differentially expressed RNAs between MRL vs. CBA, CBA vs. STR and MRL vs. STR mice. The size of each node indicates the number of connected components. The color shows the clustering coefficient distribution to identify a modular organization of networks. The clustering coefficient of a node is the number of triangles (3-loops) that pass through this node, relative to the maximum number of 3-loops that could pass through the node.

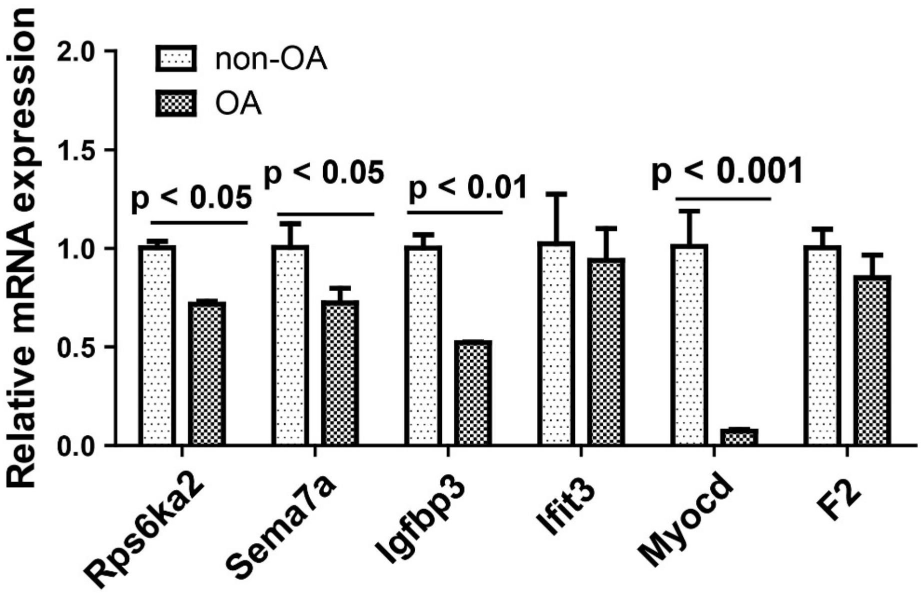

Figure s9 Relative mRNA expression analysis of candidate genes in human $CD44^{+}$ $CD90^{+}$ CSPCs isolated from patients with (OA) and without (non-OA) osteoarthritis. n = 3-5 per group.

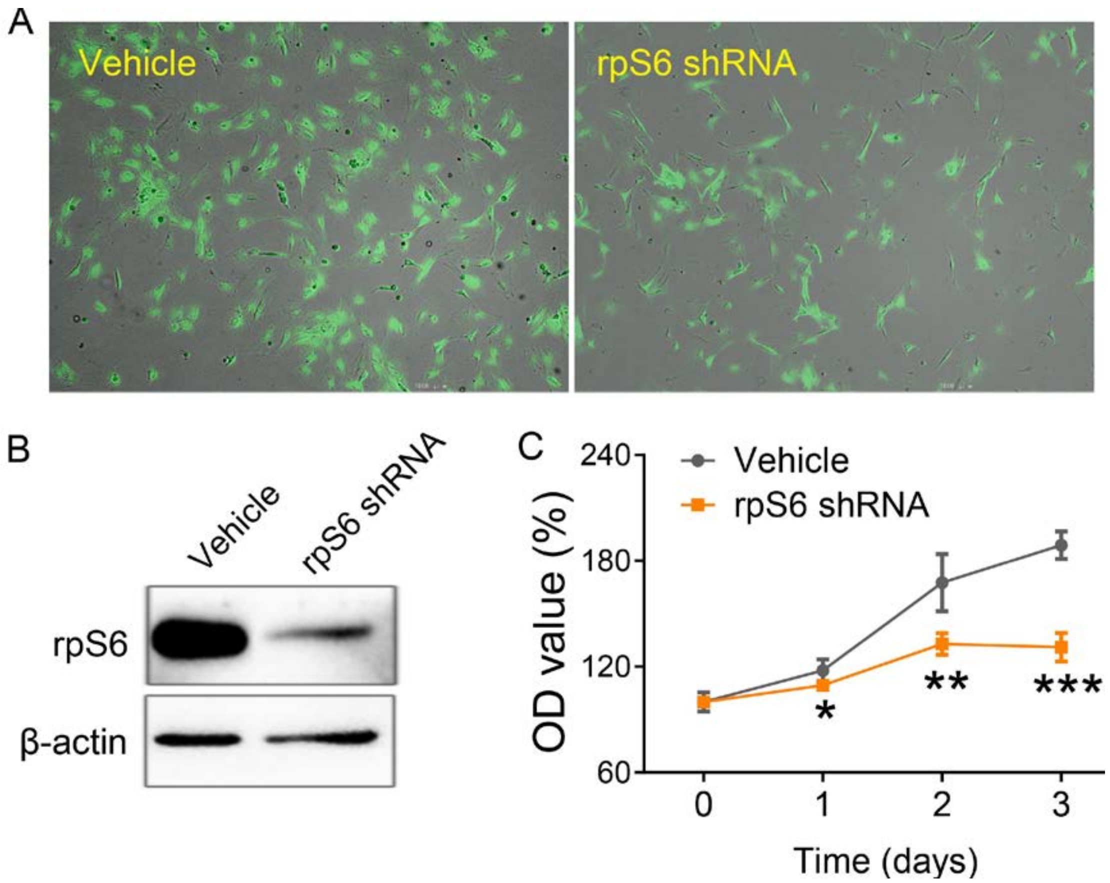

Figure s10 Decreasing rpS6 expression with shRNA inhibited the proliferation of $CD44^+$ $CD90^+$ CSPC from the CBA mice. A: GFP-positive cells indicated the success of virus infection. B: Reduced protein expression of rpS6 in CSPC after rpS6 shRNA treatment. C: Proliferative ability of CSPCs was monitored 0-3 days after culturing by CCK-8 assays. *$p < 0.05$, **$p < 0.01$, ***$p < 0.001$ versus vehicle (n = 6 per group).

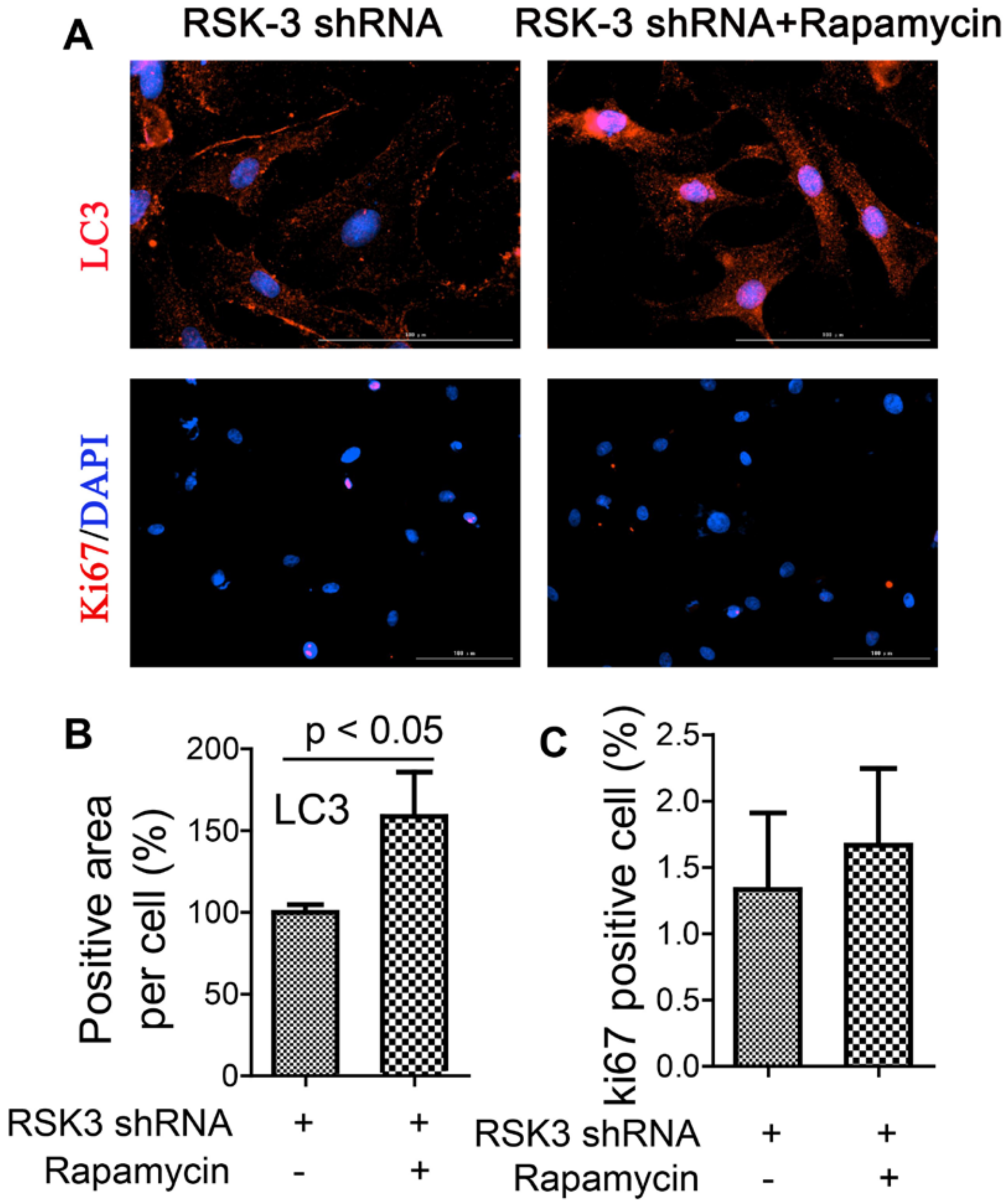

Figure s11 Ki67 and LC3 expression in CD44$^+$ CD90$^+$ CSPCs treated with shRNA to down-regulate RSK-3 and/or rapamycin to promote autophagy. Protein expression and statistical analysis of LC3 (A,B) and Ki67 (A,C). n = 6 per group.

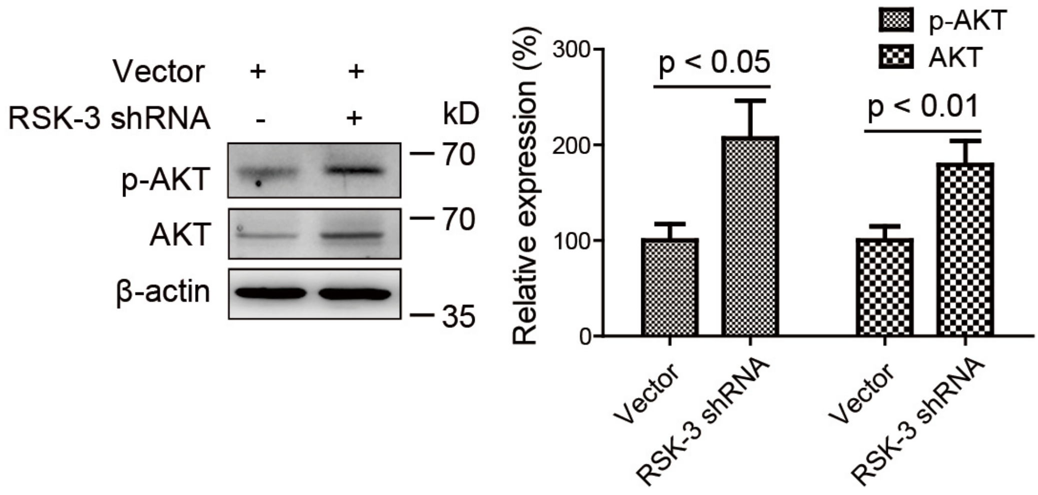

Figure s12 Effects of downregulated RSK-3 in MRL/MpJ CD44$^+$ CD90$^+$ CSPCs on protein expression of AKT. n = 6 per group.
